# High expression of RARG accelerates ovarian cancer progression by regulating cell proliferation

**DOI:** 10.3389/fonc.2022.1063031

**Published:** 2022-11-29

**Authors:** Lin Xiu, Yuxi Zhao, Ning Li, Jia Zeng, Jing Liu, Yongliang Fu, Qiao Gao, Lingying Wu

**Affiliations:** ^1^ Department of Gynecology Oncology, National Cancer Center/National Clinical Research Center for Cancer/Cancer Hospital, Chinese Academy of Medical Sciences and Peking Union Medical College, Beijing, China; ^2^ Department of Pathology, National Cancer Center/National Clinical Research Center for Cancer/Cancer Hospital, Chinese Academy of Medical Sciences and Peking Union Medical College, Beijing, China; ^3^ Centre for Medicines Discovery, Nuffield Department of Medicine, University of Oxford, Oxford, United Kingdom

**Keywords:** RARG, ovarian cancer, prognosis, cell proliferation, biomarker

## Abstract

**Purpose:**

To explore the relationship between retinoic acid receptor gamma (RARG) and ovarian cancer (OC) cell proliferation and the prognosis of patients.

**Methods:**

The transcriptome and clinical information of 379 OC and 88 normal ovarian samples were downloaded from the Cancer Genome Atlas (TCGA) database and the Genotype Tissue Expression (GTEx) database. We compared the mRNA level of RARG between ovrian normal and tumor tissues with the Wilcoxon rank sum test.The R package “limma” was used to analyze the differences in RARG expression between different clinical subgroups. Kaplan−Meier analysis was applied to evaluate the correlation between RARG and prognosis of patients. A nomogram was established to predict the effect of RARG on prognosis of OC patients. Immunohistochemistry and qRT−PCR experiments were conducted to determine the differential expression of RARG between ovarian normal and tumor tissues. Finally, we altered RARG expression using specific siRNA and lentiviral expression vectors to explore the function of RARG by CCK-8, cell cycle, colony formation, and xenograft assays in nude mice.

**Results:**

RARG was highly expressed in ovarian tumors and was an independent predictor of poor overall survival outcomes. Subgroup analysis showed the high expression of RARG was related to FIGO stage III-IV (P=0.027), overall survival time <5 years (P=0.013) and dead status (P=0.041). The Kaplan-Meier curve indicated that patients with high RARG expression level had poor prognosis. The area under the curve (AUC) of RAGR expression for predicting patient survival rates at 1, 5 and 9 years were 0.659, 0.616 and 0.627, respectively. The GSEA enrichment analysis revealed that RARG was involved in ovarian cancer progression through multiple pathways. In cellular experiments *in vitro*, downregulation of RARG expression significantly suppressed the proliferation and colony formation capacity of OC cells. In cellular experiments *in vivo*, knockdown of RARG significantly reduced tumor growth in nude mice, decreased expression levels of Ki-67 and proliferation cell nuclear antigen (PCNA).

**Conclusions:**

High expression of RARG could promote OC cell proliferation and was an independent predictor of poor prognosis. RARG might work as a potential molecular target and biomarker for individualized diagnosis and treatment in OC patients.

## Introduction

Ovarian cancer (OC) is the third most common malignant tumor of the female reproductive system (1, 2). There are approximately 225,000 new cases in the world every year (3). Although the 5-year survival rate for pateints with early-stage ovarian cancer can be varied from 60% to 90%,while advanced OC could be an insidious disease with high mortality (4). At present, effective predictive biomarkers for clinical diagnosis and treatment strategies are urgently needed.

OC is a highly heterogeneous disease, and differentiation based on stage alone is not sufficient for clinical diagnosis (5). Therefore, there is an urgent need to explore and establish a classification method combining molecular phenotypes with clinicopathological features to better guide the clinical treatment, prognosis prediction and individualized treatment of patients. To date, several targeted therapies have been used in OC treatments. For example, PARP inhibitor, the first drug targeting the DNA damage response to have entered the clinic, represent a successful example of molecular target therapy.

Retinoic acid receptors (RAR) are members of the nuclear receptor superfamily that mediate the multipotent effects of the vitamin A metabolite retinoid and its derivatives in normal and cancer cells (6, 7). RAR is encoded by three distinct genes, α, β, and γ (8). Retinoic acid receptor γ (RARG), which is 90% homologous to retinoic acid receptor α (RARA) and retinoic acid receptor β (RARB), is unique among the three RAR subtypes because it typically resides in the cytoplasm of cancer cells (9). Studies have found that RARG plays a vital role in the occurrence and development of many tumors, such as hepatocellular carcinoma, esophageal carcinoma and prostate cancer, and its expression level is closely related to the proliferation and migration ability of tumor cells (10–12). However, the role of RARG in the occurrence and development of ovarian cancer is still unclear.

Using bioinformatics and molecular biology techniques, we performed the present study to explore the function of RARG on OC and to evaluate its correlation with the prognosis of OC patients. Our study used TCGA and GTEx database to calculate RARG expression in ovarain cancer tissues and normal ovarian tissues. The function of RARG on cell proliferation, cell cycle and colony formation ability were investigated by using specific siRNA and lentiviral expression vectors in two OC cell lines. The highlight was that we further used xenograft assays in nude mice to confirm the role of RARG in OC.

## Materials and methods

### Data collection and processing

The clinical information and mRNA expression data of 379 OC patients were collected from The Cancer Genome Atlas (TCGA) database, and the gene expression profiles of 88 normal ovarian samples were obtained from the Genotype Tissue Expression (GTEx) database. Normalized gene expression was measured as fragments per kilobase of transcript per million mapped reads (FPKM), and the values were log2-transformed.

### RARG differential expression and prognosis correlation

The correlations between mRNA level of RARG and tisseus were analyzed by Wilcoxon rank sum test. Based on different clinical subgroups, the differential expression of RARG was calculated by the R package “limma”. According to the expressed median value of RARG, the OC patients were divided into a RARG-high expression group and a RARG-low expression group. Kaplan−Meier survival analysis was performed by the “Survival” R package to evaluate the difference in survival rates between the two groups. The area under time-dependent receiver operating characteristic (ROC) curve was used to evaluate the predeiction efficiency of RARG by the R package “survival ROC”.

### Construction and validation of a nomogram incorporating RARG

We sequentially conducted univariate and multivariate Cox regression analyses to identify the association of RARG with OC prognosis. To accurately and intuitively predict the prognosis of patients, a nomogram was constructed based on RARG and clinical features using the R package “rms”. The consistency between the actual survival rates and the nomogram-predicted rates was determined using calibration plots to evaluate the calibration of the nomogram.

### Functional enrichment and analysis of immune cell infiltration

We took the absolute value of correlation coefficient greater than 0.3 as the threshold to screen RARG related genes (**Supplementary Excel 1**). Based on the above genes, we performed GO enrichment analysis *via* the “clusterprofiler” package to explore the function of RARG. We screened the differentially expressed genes in RARG-high expression group and RARG-low expression group with logFC greater than 0.2 and P < 0.05. Then GSEA (www.gsea-msigdb.org/gsea/index.jsp) was utilized to assess these gene sets to determine if the identified sets had significant differences. We set |Normalized enrichment score (NSE)|>1, nominal (NOM) p value < 0.05 and FDR q-value < 0.25 as the cutoffs.

The relative tumor infiltration levels of 22 immune cell types were determined by ssGSEA. The difference in immune cell abundance between the RARG-high expression and RARG-low expression groups was analyzed by the “limma” R software package.

### Tissue specimens

20 OC tissues and 10 normal ovarian tissues were collected from patients who underwent surgical resection in National Cancer Center/National Clinical Research Center for Cancer/Cancer Hospital, Chinese Academy of Medical Sciences and Peking Union Medical College (from September 2016 to August 2017). Pathologists immediately isolated tissue from each patient and stored it in liquid nitrogen. Three pathlogists confirmed the pathological examination of ovarian cancer. Ethical approval was provided by the Ethics Committee of the Cancer Institute (Hospital), CAMS&PUMC (21/146-2817).

### Cell culture and transfection

The human OC cell lines A2780 and SKOV3 were provided by the Cell Resource Center (IBMS, CAMS/PUMC). Both cell lines were cultured in RPMI 1640 medium supplemented with 10% fetal bovine serum (Invitrogen, San Diego, CA) at 37°C with 5% CO_2_. We synthesized small interfering RNA (siRNA) against human RARG and control nonsilent siRNA (GenePharma Shanghai, China). The siRNAs were transfected with Lipofectamine 2000 (Invitrogen, San Diego, CA, USA) according to the kit instructions, and the final concentration used for RARG silencing was 50 nM. A lentiviral vector PLKO.1-shRARG-GFP knocking out RARG was constructed by GenePharma. To generate the lentivirus, 293FT cells were cotransfected with psPAX2, pMD2G and PLKO.1-shRARG-GFP. 48 hours after transfection, the lentiviral supernatants were collected and filtered through a 0.22 μm filter. The filtered lentivirus was incubated with polybrene (Sigma, St. Louis, MO, USA) and transfected into ovarian cancer cells, and then the cells were screened with puromycin (0.8 μg/ml, Invitrogen) for 72 hours. The sequences of siRNAs and shRNAs are listed in **Table 1**.

**Table 1:** The sequences of siRNAs and shRNAs.

Groups	Sequences
Non-silencing	5’-TTCTCCGAACGTGTCACGT-3′
RARG –siRNA-1	5’-CCCTCAGTTAGAAGAGCTCAT-3’
RARG –siRNA-2	5’-GCTGCGTATCTGCACAAGGTA-3’
RARG -shRNA	5’-CAATGACAAGTCCTCTGGCTA-3’

### Total RNA extraction, quantitative real-time PCR, western blot and immunohistochemistry

The samples for qRT-PCR was extracted from 10 pairs of OC tissues and adjucent normal tissues frozen in liquid nitrogen with TRIzol reagent (Invitrogen, Carlsbad, CA, USA). Following the manufacturer’s instruction, total RNA was isolated using an RNApure Tissue & Cell Kit (Cwbiotech, Beijing, China). The isolated RNA was used as a template for reverse transcription reactions using a HiFiScript cDNA Synthesis Kit (Cwbiotech). Quantitative real-time RT−PCR (qRT-PCR) analysis was performed using SYBR^®^ Fast qPCR Mix (TaKaRa, Shiga, Japan) and a CFX96 Real-Time System (Bio-Rad). The primer sequences are shown in **Table 2**. GAPDH served as an internal control. Each samples were performed independently for three times.

**Table 2:** Primers for qRT−PCR analysis.

Gene	Primer	Sequence
GAPDH	Forward	5’-CAGCCTCAAGATCATCAGCA-3′
	Reverse	5’-TGTGGTCATGAGTCCTTCCA-3′
RARG	Forward	5’-AACAAGGTGACCAGGAATCG-3′
	Reverse	5’-TGTCAGGTGACCCTTCTTCC-3′

Total protein was isolated from tissue samples by using RIPA buffer (Applygen, Beijing, China) with protease inhibitors (Roche, Basel, Switzerland). Immunoblotting was performed with primary antibodies against RARG (1:1000, Proteintech, Wuhan, China). GAPDH (1:5000, Proteintech) was used as a loading control. Secondary antibodies (goat anti-mouse IgG and goat anti-rabbit IgG, 1:5000) were purchased from Applygen. The signals were visualized with a super enhanced chemiluminescence (ECL) detection reagent (Applygen).

For immunohistochemistry, a total of 20 OC tissues and 10 normal ovarian tissues that were embedded in paraffin were collected from patients who underwent surgical resection in CAMS hospital, China (from September 2016 to August 2017). 4-μm-thick sections of paraffin-embedded tissue arrays were deparaffinized in xyleme, rehydrated in graded ethanol and incubated in 3% hydrogen peroxide in methanol to block endogenous peroxidase. The sections were then heated for 20 min in 0.01 mol/l citrate buffer (pH 6.0) in a microwave oven. After washing the sections with PBS 3 times, they were incubated with an anti-human RARG antibody (1:500 dilution; Proteintech, Wuhan, China) at 4°C overnight. After washing in PBS, the sections were developed according to the manufacturer’s instructions (PV-9000 Polymer Detection System, ZhongShan Golden Bridge, China), counterstained with hematoxylin, dehydrated in graded ethanol and sealed with neutral resin. For immunohistochemistry in the xenografts resected from nude mice, experimental process was similiar with primary antibodies including anti-RARG antibody (1:500 dilution; Proteintech), anti-Ki67 antibody (1:200 dilution; Abcam) and anti-Proliferating Cell Nuclear Antigen (PCNA; 1:300 dilution; CST). Two pathologists used 200x light microscopy to evaluate the immunostaining.

### Cell viability assay and colony formation assay

According to the manufacturer’s protocol, the Cell Counting Kit-8 assay (CCK-8, Dojindo, Japan) was used to evaluate the effect of RARG on cell proliferation of A2780 and SKOV3. Each well seeded 1×10^3^ transfected cells in 96-well plates. For 7 consecutive days, adding 10μL CCK8 solution to each well every 24 h. After incubating in 37°C with 5% CO_2_ for 1h, the absorbance at 450 nm measured by an automatic microplate reader (BioTek, Winooski, VT, USA) was used to read the optical density (OD) value. The relative cell viability (%) was finally calculated.

After RARG knockdown, 500 cells were seeded in each well of a six-well plate to evaluate the effect of RARG on cell clonogenesis. The cells were grown for 10-14 days to form colonies, which were then fixed with methanol and stained with crystal violet. The colonies were counted.

### Cell cycle analysis

The cell cycle distribution of 1.0×10^6^ RARG-knockdown cells and 1.0×10^6^ negative control cells were fixed with 70% ethanol at 4°C overnight. The cells then were resuspended and analyzed by a cell cycle detection kit (KeyGen Biotech, Nanjing, China) and BD™ LSRII flow cytometry (BD Biosciences, San Jose, CA, USA). The cell cycle curves were analyzed by ModFit software (Verity Software House, Topshem, ME, USA).

### Xenograft model antitumor assay

Animal experiments were approved by the Animal Center of the National Cancer Center/Cancer Hospital Research Institute of the Chinese Academy of Medical Sciences (NCC2021A-002). Ten 4-weeks-old female nude mice (BALB/C-NU, HFK Bioscience, Beijing, China) were randomly divided into two groups. All mice were raised in a special conditions under 12/12 cycle of light with 25-27°C temperature. Feed all mice with enough food and water as animal’s feeding guideline. A total of 3×10^7^ sh-RARG A2780 cells and 3×10^7^ sh-Scramble A2780 cells were injected subcutaneously into both groups. The tumor volume V was calculated by measuring the tumor size every 3 days. The formula was as follows: V= π/6×L×W^2^ (L: the length of the tumor, W: the width of the tumor). At the end of the experiment, used inhalational anesthesia with 2% isoflurane to sacrifice the mice, and followed by cervical dislocation. The tumor tissues were further resected and weighed. To comfirm the differences in the protein expression levels of RARG, ki-67 and PCNA between sh-RARG group and sh-Scramble group, the tumor tissues were embedded in paraffin and sectioned for immunohistochemistry.

### Statistical analysis

All statistical analyses were performed by R software 3.5.3 and SPSS 22.0 software. Chicago, IL, USA). All expereimet data were performed mean ± standard deviation. Differences between two groups were analyzed by Student’s t-test, and differences between three or more groups were analyzed by ANOVA. The survival analysis was performed using Kaplan-Meier curves, and the differences between curves were verified using the log-rank test for OS. The independent prognostic factors were analyzed by the univariate and multivariate Cox regression models. P < 0.05 was considered statistically significant.

## Results

### The mRNA level of RARG was highly elevated in tumor tissues of OC patients

**Figure 1:**
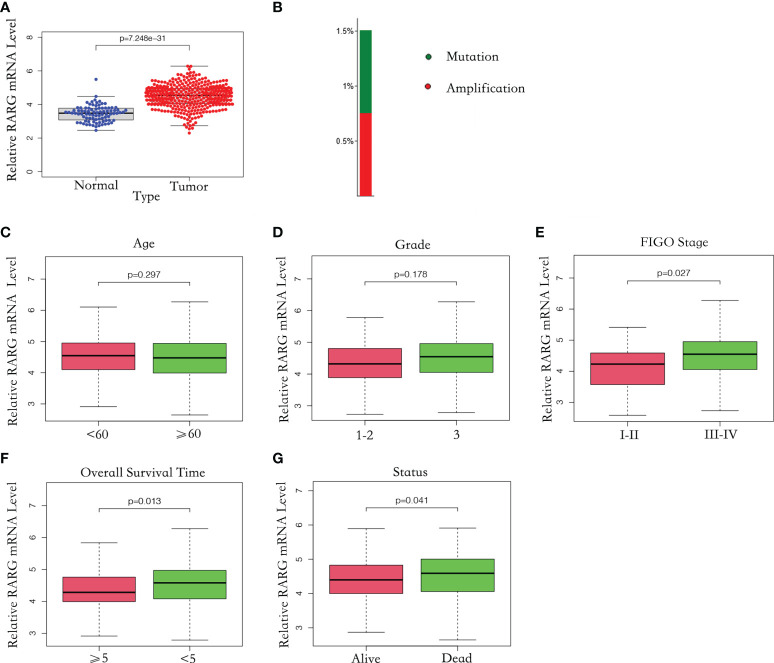
The mRNA level of RARG was abnormally elevated in OC. **(A)** Abnormally high mRNA level of RARG in OC. **(B)** RARG gene alterations in OC. **(C-G)** The association of the RARG mRNA level with age **(C)**, grade **(D)**, stage **(E)**, OS time **(F)** or status **(G)**.

Firstly, we compared RARG mRNA level in 88 normal ovarian samples from the GTEx dataset with that in 379 OC samples from the TCGA dataset. The clinical characteristics and RARG expression data of 379 primary OC patients were shown in **Table 3**. The median age at diagnosis was 59 years old. In our study cohort, 0.26% of the tumors were well differentiated, 11.85% were moderately differentiated, and 85.20% were poorly or not differentiated. There was 1 patient (0.26%) of stage I primary OC, 23 patients (6.06%) of stage II OC, 295 patients (77.84%) of stage III OC, and 57 patients (15.04%) of stage IV OC. Of the subjects who were alive at the last follow-up, the median survival time was 797 days (range: 8-5481 days). The mean RARG of normal ovarian samples and OC samples were 3.11 and 4.27, respectively. The results demonstrated that compared with normal ovarian samples, RARG was significantly overexpressed in OC cells (p<0.001, **Figure 1A**). Second, we searched for gene alterations of RARG on the cBioPortal website (**Figure 1B**). It revealed that 1.5% of patients with OC had RARG gene alterations, including amplification and gene mutations.

**Table 3:** Clinical characteristics of the 379 OC patients.

Characteristics	Number of sample size (%)
Age (years)	
<60	199 (52.51)
>=60	180 (47.49)
Histologic grade	
Grade 1	1 (0.26)
Grade 2	45 (11.85)
Grade 3	322 (84.94)
Grade 4	1(0.26)
NA	10 (2.6)
TNM Stage	
Stage I	1 (0.26)
Stage II	23 (6.06)
Stage III	295 (77.84)
Stage IV	57 (15.04)
NC	3 (0.80)
Therapy outcome	
Complete remission	213 (56.20)
Partial remission	43 (11.35)
Progressive disease	27 (7.12)
Stable disease	22 (5.80)
NA	74 (19.53)
Vital status	
Living	147 (38.79)
Deceased	232 (61.21)

NA not available.

### RARG mRNA levels in different clinical subgroups

To explore the correlation between RARG and clinicopathological parameters, we excluded patients with unknown tumor stage, tumor grade and treatment outcomes. A total of 362 OC samples with complete RARG expression data and clinicopathological characteristics from the TCGA dataset were analyzed. As shown in **Figure 1C, D**, there was no significant difference in RARG mRNA levels between subgroups of age (p = 0.297) and grade (p = 0.178). However, the RARG mRNA level was correlated with the pathologic stage of OC patients, the mean value of RARG in stageIII-IV patients and stageI-IIpatients were 4.64 and 4.27, which the level in stageIII-IV patients was higher than that in stageI-IIpatients (**Figure 1E**, p<0.05). The RARG mRNA level in patients with survival time less than or equal to 5 years was higher than that in patients with survival time greater than 5 years (**Figure 1F**, p<0.05). The RARG mRNA level in patients who died was higher than that in patients who survived (**Figure 1G**, p<0.05).

### Elevation of RARG mRNA level was positively correlated with poor prognosis

**Figure 2:**
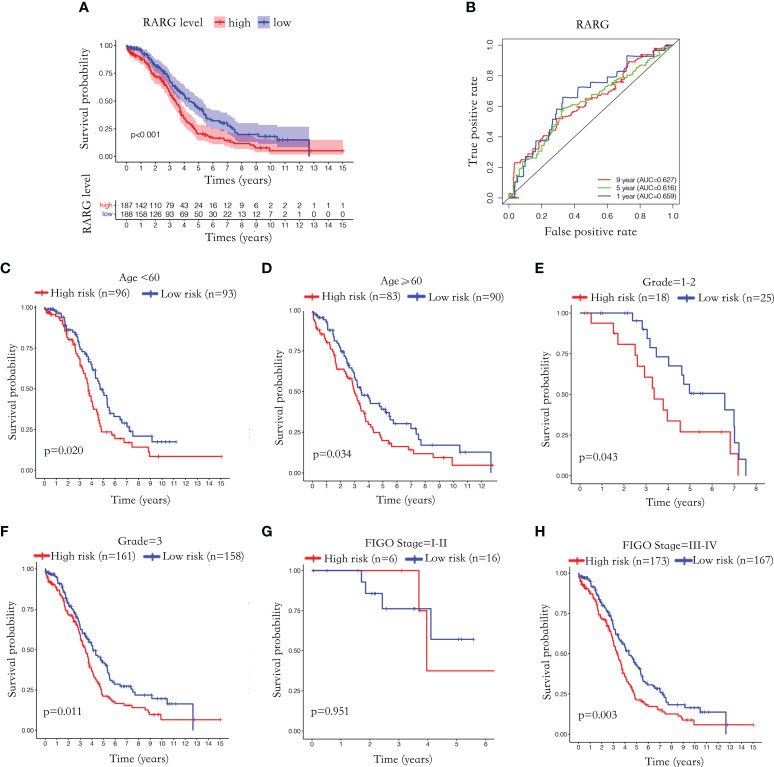
Abnormal elevation of RARG mRNA level was positively correlated with poor prognosis. **(A)** The Kaplan-Meier survival curves of the RARG high expression group and the low expression group. **(B)** ROC curves were used to assess the efficiency of RARG for predicting 1, 5 and 9 year survival. **(C–H)** The Kaplan-Meier survival curves of RARG high expression group and low expression group in the age<60 subgroup **(C)**, age≥60 subgroup **(D)**, grade 1-2 subgroup **(G)**, grade 3 subgroups **(H)**, stage I-II subgroup **(E)**, stage III-IV subgroup **(F)**.

To further explore the relationship between RARG mRNA level and prognosis of OC patients, we excluded patients with unknown prognostic information and 375 OC patients were enrolled. Based on the median value of RARG expression, 375 OC patients were divided into 187 RARG-high expression patients and 188 RARG-low expression patients. The Kaplan−Meier survival curves indicated that patients with high RARG expression had poorer prognosis (**Figure 2A**, p < 0.001). The prognostic assessment effect of RARG in OC samples was analyzed by ROC curve analysis. The areas under the curve (AUCs) of RARG for predicting 1, 5 and 9 year survival were 0.659, 0.616 and 0.627, respectively (**Figure 2B**).

To further evaluate the prognostic predictive effect of RARG, we performed Kaplan−Meier survival analyses based on different clinical subgroups. The Kaplan−Meier survival curve showed that patients with high RARG expression in the age <60 (**Figure 2C**, p < 0.05), age ≥60 (**Figure 2D**, p < 0.05), grade 1-2 (**Figure 2E**, p < 0.05), grade 3 (**Figure 2F**, p < 0.05) and stage III-IV (**Figure 2H**, p < 0.01) subgroups had a poor prognosis. In stage I-II subgroup, high RARG expression had no statistically significance with poor prognosis (**Figure 2G**, p > 0.05). These results indicated that high expression of RARG is positively associated with poor prognosis in most clinical subgroups, suggesting that elevated RARG might be a good biomarker for predicting the prognosis of OC patients.

### Establishment and validation of a nomogram based on RARG

**Figure 3:**
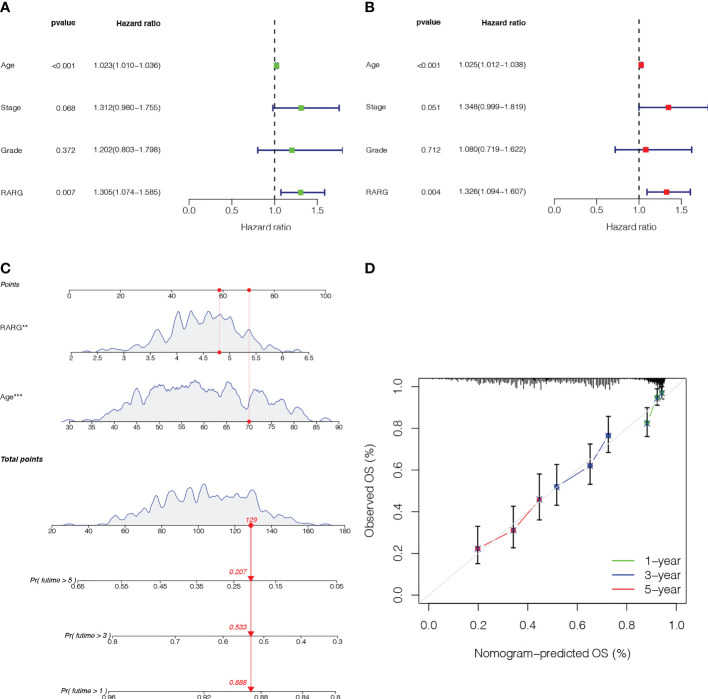
Establishment and validation of a nomogram based on the RARG. **(A)** Univariate Cox regression analysis. **(B)** Multivariate Cox regression analysis. **(C)** A nomogram was constructed for predicting the 1-, 3- and 5-year survival rates of OC patients. **(D)** Calibration curves showed the actual rate versus predicted probability of 1-, 3- and 5-year survival.

We performed univariate Cox regression and multivariate Cox regression to assess the independent prognostic value of clinicopathological parameters (age, stage and grade) and RARG derived from the TCGA set (**Figures 3A, B**). The multivariate Cox regression analysis showed that the HR value of RARG was 1.326 (**Figure 3B**, p < 0.01), which indicated that RARG was an independent risk factor for the prognosis of OC patients.

To provide an accurate classifier for predicting the prognosis of OC patients, we constructed a nomogram incorporating RARG and age (**Figure 3C**). The results showed that the calibration curves for 1-, 3- and 5-year survival rates were close to the standard curves, indicating that the nomogram had good prediction performance (**Figure 3D**).

### Functional enrichment analysis of RARG in OC

**Figure 4:**
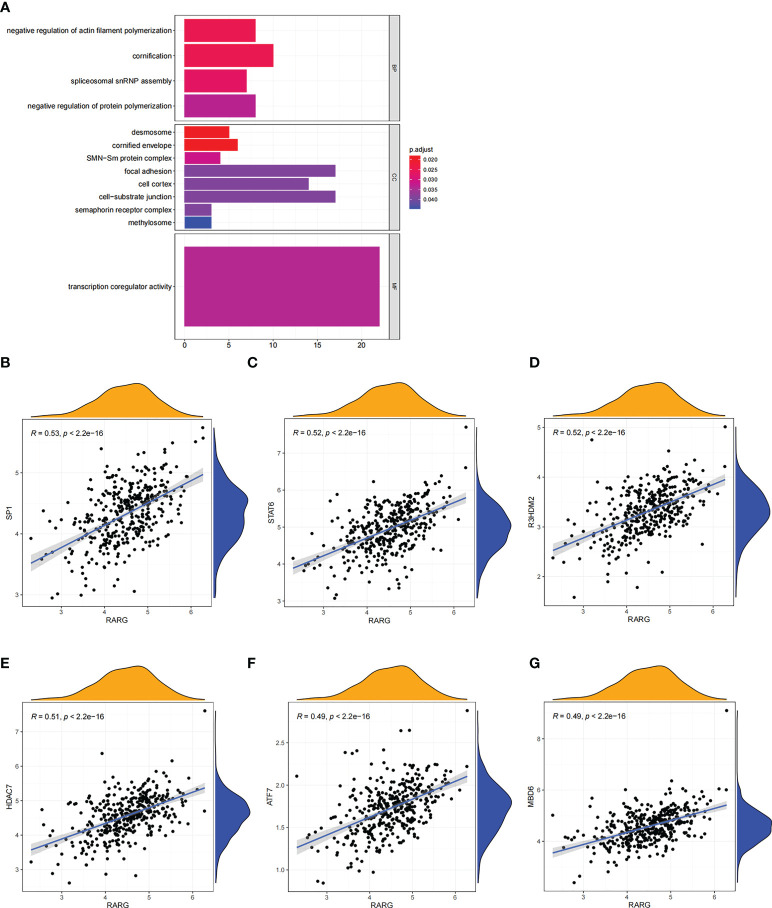
GO enrichment analysis of RARG related genes. **(A)** The results of GO enrichment analysis of RARG related genes were shown in a bubble chart: molecular function (MF), biological process (BP), and cellular component (CC). **(B–G)** RARG was significantly correlated with SP1 **(B)**, STAT6 **(C)**, R3HDM2 **(D)**, HDAC7 **(E)**, ATF7 **(F)** and M8D6 **(G)**.

**Figure 5:**
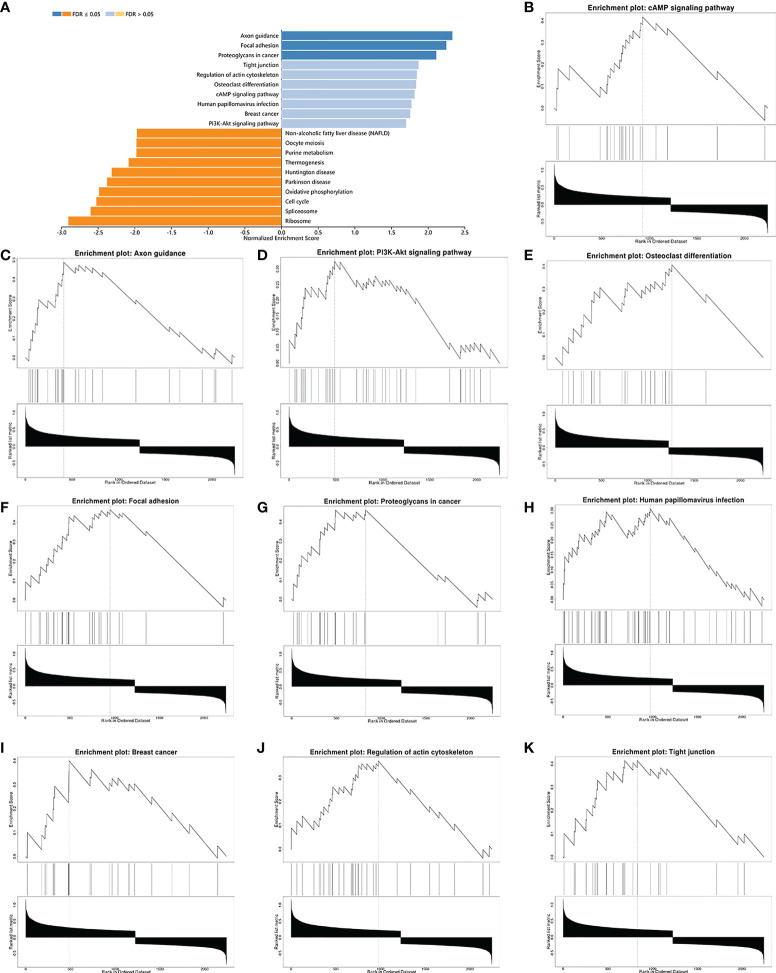
GSEA enrichment analysis of differential genes in high and low RARG expression groups. **(A)** Histogram of GSEA enrichment of differentially expressed genes in high and low RARG expression groups. **(B–K)** The top 10 biological pathways positively correlated with high RARG expression.

Subsequently, gene-ontology (GO) enrichment analysis was performed for RARG related genes (**Figure 4A**). The biological process of GO enrichment included negative regulation of protein polymerization and the molecular function of enrichment included transcription coregulator activity. As shown in **Figures 4B–G**, the most related genes of RARG were SP1 (p < 0.001), STAT6 (p < 0.001), R3HDM2 (p < 0.001), HDAC7 (p < 0.001), ATF7 (p < 0.001) and M8D6 (p < 0.001). Subsequently, we performed GSEA enrichment analysis on genes differentially expressed between high and low RARG expression groups. The results of GSEA enrichment analysis showed that cAMP signaling pathway, Axon guidance, PI3K-Akt signaling pathway, Osteoclast differentiation, Focal adhesion, Proteoglycans in cancer, Human papillomavirus infection, Breast cancer, Regulation of actin cytoskeleton and Tight junction were positively correlated with the high expression of RARG (**Figures 5A–K**), while Non-alcoholic fatty liver disease (NAFLD), Oocyte meiosis, Purine metabolism, Thermogenesis, Huntington disease, Parkinson disease, Oxidative phosphorylation, Cell cycle, Spliceosome and Ribosome were negatively correlated with the high expression of RARG (**Supplementary Figures 1A–J**). The results indicated that the progression of ovarian cancer can be regulated through multiple pathways by high expression level of RARG.

### Analysis of immune cell infiltration in high and low RARG expression group

**Figure 6:**
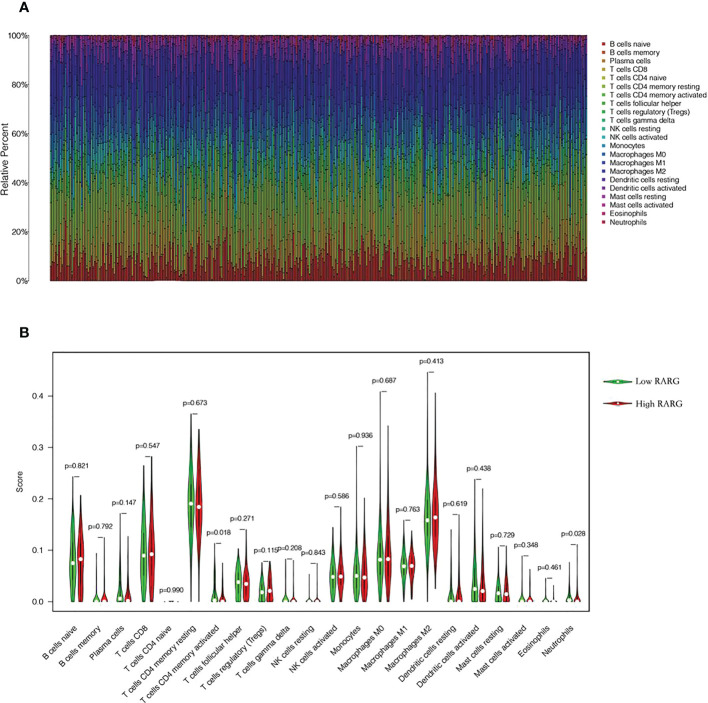
Analysis of immune cell infiltration in high and low RARG expression group. **(A)** Infiltration abundance of 22 immune cells in each OC sample. **(B)** The differences in the infiltration degree of 22 immune cells between the RARG low expression and high expression groups. The green line represents RARG low expression group, and the red line is the RARG high expression group.

To explore the differences in immune cell infiltration between high and low RARG expression groups, we first mapped the immune cell infiltration of each ovarian cancer patient in the TCGA cohort (**Figure 6A**). The infiltration degree of CD4 memory activated T cells (**Figure 6B**, p < 0.05) and Neutrophils (**Figure 6B**, p < 0.05) in RARG low expression group was higher than that in RARG high expression group.

### Downregulation of RARG inhibited OC cell proliferation *in vitro* and *in vivo*

**Figure 7:**
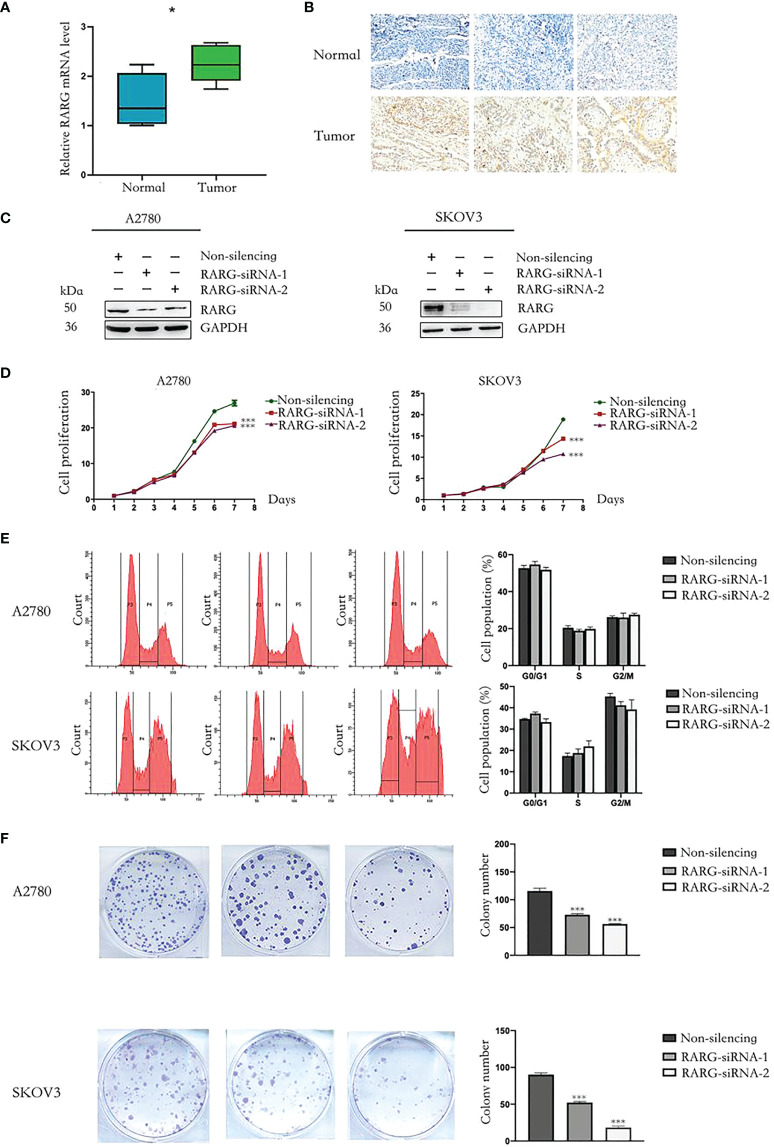
Downregulation of RARG inhibited proliferation of OC cells. **(A)** The qRT-PCR analysis of RARG mRNA levels in ovarian tumor tissues and adjacent normal tissues. **(B)** Immunohistochemistry analysis of RARG protein levels in ovarian tumor tissues and adjacent normal tissues. **(C)** Western blotting analysis of RARG expression in A2780 cells or SKOV3 cells transfected with RARG siRNA or non-silencing siRNA. **(D)** Cell proliferation was detected by the CCK-8 assay. **(E)** Cell cycle assay. **(F)** Cell Colony assay. *P < 0.05; ***P < 0.001.

We applied qRT−PCR and immunohistochemistry to additionally comfirm the differences in the mRNA expression levels and protein expression levels of RARG between ovarian cancer tissues and adjacent normal tissues. We used qRT−PCR which total RNA was extracted from 10 pairs of OC tissues and adjucent normal tissues to investigate the articulation verbalization of RARG at the mRNA transcriptional level. The results showed mRNA expression levels of RARG in ovarian cancer tissues were higher than adjacent normal tissues (**Figure 7A**). We further used immunohistochemistry to measure the RARG protein levels obtained from OC tissues and adjacent normal tissues. The results showed that RARG protein levels were higher in OC tissues than that in adjacent normal tissues (**Figure 7B**).

To clarify the functional role of RARG in OC cells, we applied RARG-siRNA to reduce its protein expression levels both in A2780 and SKOV3 cells (**Figure 7C**). Cell proliferation was detected by CCK-8 assay. The downregulation of RARG expression significantly suppressed the proliferation (**Figure 7D**, p < 0.001) and colony formation capacity of A2780 cells and SKOV3 cells (**Figure 7F**, p < 0.001). Cell cycle was not significantly different between the RARG siRNA group and the negative control group (**Figure 7E**). Collectively, theses data revealed that RARG could facilitate the proliferation and colony ability of OC cells *in vitro*.

**Figure 8:**
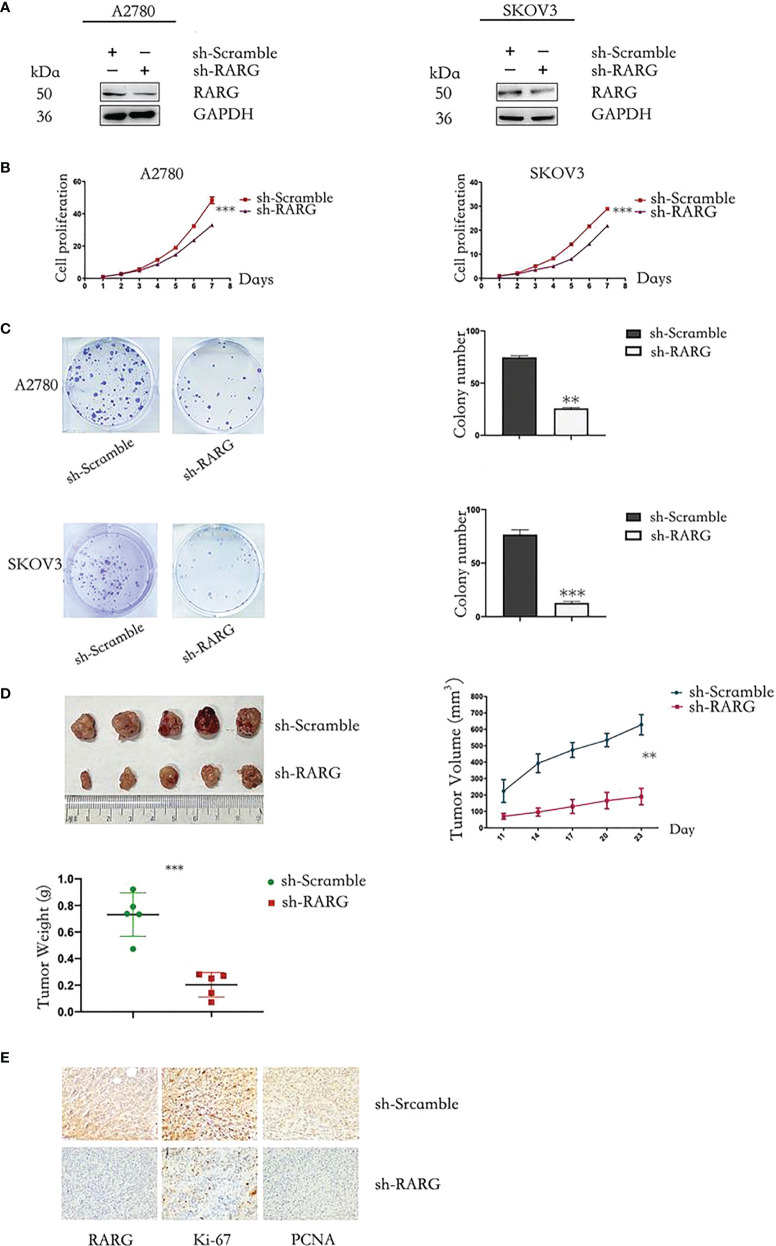
Knocking out RARG inhibited proliferation of OC cells. **(A)** Western blotting analysis of RARG expression in A2780 cells or SKOV3 cells transfected with RARG shRNA or scramble shRNA. **(B)** Cell proliferation was detected by the CCK-8 assay. **(C)** Cell Colony assay. **(D)** The xenograft tumors, tumor growth curve and weight of xenograft tumors. **(E)** Immunohistochemistry analysis of RARG, Ki-67 and PCNA protein levels in sh-Scramble group and sh-RARG group. Scale bar = 1 cm. **P < 0.01; ***P < 0.001.

In addition, western blot assay showed that sh-RARG stably knocked out RARG protein expression in A2780 cells and SKOV3 cells (**Figure 8A**). The CCK-8 assay results showed that the sh-RARG reduced the proliferation (**Figure 8B**, p < 0.001) and colony formation capacity (**Figure 8C**, p < 0.001) of A2780 cells and SKOV3 cells compared with the sh-Scramble group. Furthermore, in order to clarify the promoting effect of RARG on ovarian cancer cells *in vivo*, we used sh-RARG group (n=5) and sh-Scramble group (n=5) of A2780 cells to inoculate nude mice subcutaneously. Each mouse was inoculated with 4 million cells and successfully constructed the xenografted tumor mouse model. The formation of measurable tumors in nude mice was first detected on the 11th day after tumor cell inoculation, which was counted as the first day of measurement. In subsequent experiments, the body weight and volume of nude mice were counted every 3 days. On the 19th day of measurement, the mean volume of transplanted tumors in the sh-RARG group and sh-Scramble group were 190.400mm^3^ and 627.600mm^3^, respectively. The volume of transplanted tumors in the sh-RARG group was significantly reduced compared with that in the sh-Scramble group. Later, we dissected and weighed the transplanted tumor in nude mice. It was found that the average transplanted tumor in the sh-RARG and sh-Scramble group were 0.271g and 0.738g. The weight of transplanted tumor also decreased significantly after RARG knockout. The changes in tumor volume and weight in the nude mice are shown in **Figure 8D**. The results showed that the volume and weight of transplanted tumors were significantly reduced after RARG knockout. The immunohistochemistry results showed that compared with the sh-Scramble group, protein expression levels of RARG, Ki-67 and PCNA were significantly decreased in the sh-RARG group (**Figure 8E**).

## Discussion

OC is one of the most common malignancies worldwide. OC has an insidious onset and lacks effective early screening and typical clinical symptoms, so approximately 70% of patients are already in an advanced stage at the time of diagnosis (13). In addition, OC easily invades and metastasizes, is difficult to cure by surgery and easily relapses after surgery (14). Tumor resection and postoperative platinum (cisplatin) combined with paclitaxel chemotherapy is the standard treatment for OC. However, chemotherapy not only easily produces drug resistance but also causes serious toxicity and side effects in patients, resulting in poor clinical treatment effects and poor prognosis (15). At present, a large number of molecules have been detected and identified as biomarkers related to the development and prognosis of OC. Clinically, the serological detection marker CA125 is often used in the diagnosis, efficacy evaluation and recurrence monitoring of OC, but the sensitivity and specificity of the diagnosis of early ovarian cancer is low, and it is susceptible to a variety of factors (16). Human testosterone protein 4 (HE4) can be used in the early diagnosis of OC with high sensitivity and specificity, but it still needs further clinical validation (17). Therefore, there is an urgent need to find more reliable biomarkers for the early diagnosis and targeted therapy of OC.

In this study, bioinformatics analysis showed that the mRNA level of RARG was increased in ovarian cancer tissues, and high expression of RARG was an independent predictor of poor prognosis, suggesting that RARG could be used as a molecular marker for the diagnosis and prognosis of OC. GO enrichment analysis of RARG-related genes showed that RARG may mediate transcription coregulator activity molecular functions, and the most relevant genes included SP1,STAT6,ATF7 and HDAC7. It suggested that RARG might affect the progression of ovarian cancer cells by participating in transcriptional regulation. Cell biology experiments showed that RARG knockdown attenuated the proliferation and clonogenesis of ovarian cancer cells *in vitro*, and knocking out RARG protein expression significantly inhibited the growth of ovarian tumor tissue *in vivo*. Notely, RARG can be used not only as a prognostic biomarker, but also may be a molecular target for targeted therapy of OC.

RARG is also closely related to the occurrence and development of other tumors (18). RARG can act as an oncogene or a tumor suppressor gene and plays different roles in different tumors. On one hand, RARG is significantly overexpressed in hepatocellular carcinoma, esophageal carcinoma, cholangiocarcinoma, colon cancer and other human cancers and plays an important role in promoting tumor progression through the PI3K/Akt, NF-κB, Wnt/β-catenin and other signaling pathways (19–23). On the flip side, RARG can also be a tumor suppressor that inhibits the proliferation and invasion of cancer cells. RARG can inhibit the invasive ability of melanoma by regulating the expression of carbowater transferase 10 (24). Loss of RARG expression can promote v-Ha-Ras-induced squamous cell carcinoma (25). Recent studies have reported that the expression of RARG is decreased in colorectal cancer tissues, suggesting that RARG could be used as a tumor suppressor gene to regulate the metastasis of colorectal cancer by inhibiting the Hippo-YAP signaling pathway (26). In addition, Brown G was reported RARG is selectively expressed in hematopoietic stem cells and their immediate progeny (27); RARG knockout mice display markedly reduced numbers of HSCs, which is critical for maintaining a balance between hematopoietic stem cell self-renewal and differentiation (28). RARG activation sensitizes human myeloma cells to carfilzomib treatment through the OAS-RNase L innate immune pathway (29). Researchers have developed small molecule inhibitors of RARG, the RARG selective antagonist AGN205728 can induce apoptosis through the mitochondria dependent pathway (12, 30). In conclusion, as an effector molecule, RARG plays an important role in ovarian cancer tumor cell proliferation and immune regulation. This study is also the first to report that RARG can act as an oncogene to promote ovarian cancer progression.

This study improves our understanding of the clinico-pathological significance and molecular pathogenesis of OC, we also need to conduct further research and deeply explore the mechanism of RARG in promoting the proliferation of ovarian cancer cells. Futuer work should be emphasized on studying more tissue samples to verify the bioinformatics results and developing small-molecule inhibitors to explore RARG for translational research.

## Conclusion

In summary, we found that RARG was overexpressed in OC tissues and was an independent prognostic indicator for OC. The results demonstrated that elevated RARG could effectively accelerated cell proliferation and colony ability of OC cells *in vitro* and *vivo*. Our study indicateed that RARG may become a biomarker and therapeutic target for individualized diagnosis and treatment of OC in the future.

## Data availability statement

The original contributions presented in the study are included in the article/**Supplementary Material**. Further inquiries can be directed to the corresponding author.

## Ethics statement

The studies involving human participants were reviewed and approved by Ethics Committee of the Cancer Institute (Hospital), CAMS&PUMC (2021020814242902). The patients/participants provided their written informed consent to participate in this study. The animal study was reviewed and approved by Animal Center of the National Cancer Center/Cancer Hospital Research Institute of the Chinese Academy of Medical Sciences (NCC2020C-462).

## Author contributions

LX was responsible for the design and critical revision of the article. YZ modified and polished the description of the article. NL completed the data collection, and JL helped to draft and critically revise this report. JZ was responsible for data analysis. YF and QG analyzed the newly added experimental data. LW prepared the figures and tables. All authors contributed to the article and approved the submitted version.

## Funding

Beijing Hope Run Special Fund of Cancer Foudation of China (LC2020A10).

## Acknowledgments

The authors acknowledge the transparency of the program and all those who have worked and continue to work on it.

## Conflict of interest

The authors declare that the research was conducted in the absence of any commercial or financial relationships that could be construed as a potential conflict of interest.

## Publisher’s note

All claims expressed in this article are solely those of the authors and do not necessarily represent those of their affiliated organizations, or those of the publisher, the editors and the reviewers. Any product that may be evaluated in this article, or claim that may be made by its manufacturer, is not guaranteed or endorsed by the publisher.

## Supplementary material

Supplementary Figure 1 The top 10 biological pathways negtively correlated with high RARG expression.
