# Supplementary figures and images for: High expression of RARG accelerates ovarian cancer progression by regulating cell proliferation
Source: Front Oncol. 2022 Nov 29;12:1063031. doi: 10.3389/fonc.2022.1063031 (PMC9746340; doi:10.3389/fonc.2022.1063031)

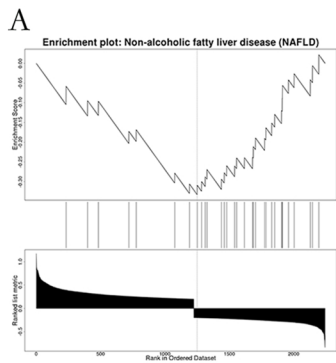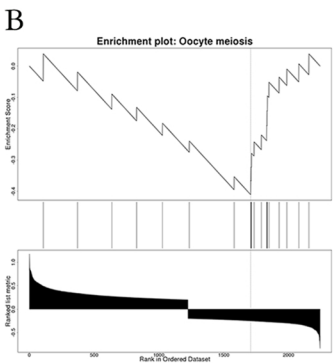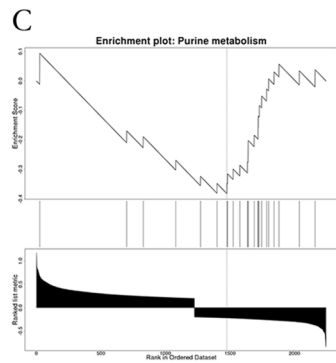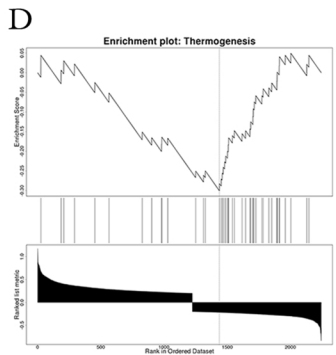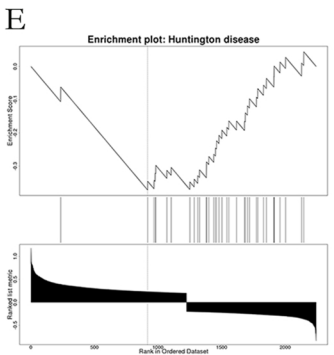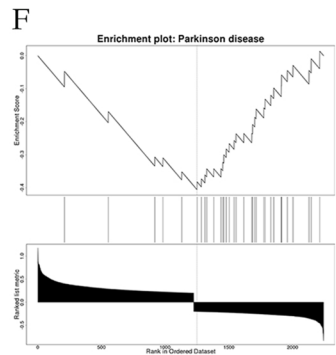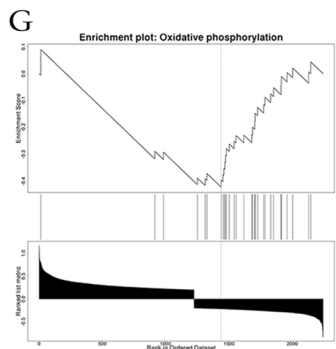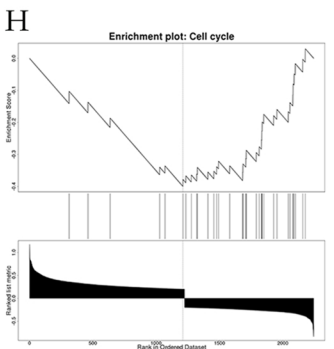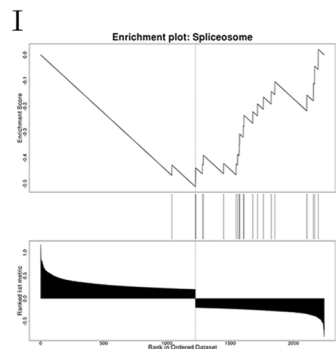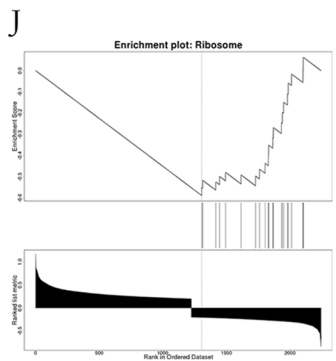

Supplement: Supplementary Figure 1 — The top 10 biological pathways negtively correlated with high RARG expression. [file DataSheet_1.zip › supplematary files/supplement figure 1.pdf]
